# Increasing Efficacy of Thrombectomy by Using Digital Subtraction Angiography to Confirm Stent Retriever Clot Integration

**DOI:** 10.7759/cureus.559

**Published:** 2016-04-04

**Authors:** Scott Simon, Sara Langan, Jonathon Cooke

**Affiliations:** 1 Department of Neurosurgery, Penn State Hershey Medical Center; 2 Naval Hospital Okinawa Department of Neurosurgery, US Navy

**Keywords:** stroke, thrombectomy, stent

## Abstract

Physicians performing thrombectomy for acute stroke have had increasing success as thrombectomy-specific devices have continued to evolve. As the devices evolve, so too must the techniques. The current generation of stent retriever thrombectomy devices requires five minutes of dwell time, regardless of the particularities of the case. We have noticed the presence of flow through the stent immediately prior to removal portends a lower chance of successful thrombus retrieval than when no flow is seen, regardless of dwell time. We hypothesize that interventionalists can use the presence or absence of flow to predict adequacy of seating time and decrease the number of deployments per case. This could significantly decrease time to recanalization by avoiding time-consuming, unsuccessful pulls. This is a technical report of a few cases of stent retriever thrombectomy. We propose using post-deployment digital subtraction angiography to confirm thrombus-device integration and increase the chance of thrombus removal.

## Introduction

Despite advances in interventional therapies, ischemic stroke remains a significant cause of morbidity and mortality in the developed world. Vessel recanalization has been acknowledged as the goal of treatment and earlier recanalization correlates with better outcomes [1-2]. First generation thrombectomy devices achieved recanalization rates of 20-40% [3-4]. The second generation of mechanical interventional devices, including Solitaire™ (Covidien, Irvine CA) and Trevo® (Stryker, Kalamazoo MI), showed recanalization rates of 69% and 86%, respectively, and have set the standard of care for mechanical thrombectomy [5-9]. As stent-retriever thrombectomy evolves, so have deployment techniques. Recommendations are to seat the device in the clot for five minutes before attempting removal. This allows adequate time for the clot to embed or seat into the cells of the stent. However, many removal attempts or “pulls” are not successful. The mean number of pulls in several large device trials ranged from 1.7 - 2.4 [4, 9-10]. A five minute seating time that does not result in success may significantly delay time to recanalization and negatively affect the outcome because the device will have to be replaced and another pull attempted. We have noticed the presence of flow through the stent immediately prior to removal portends a lower chance of successful thrombus retrieval than when no flow is seen, regardless of dwell time. We hypothesize that interventionalists can use the presence or absence of flow to predict adequacy of seating time and decrease the number of deployments per case. This could significantly decrease time to recanalization by not wasting time on unsuccessful pulls. We propose using post-deployment digital subtraction angiography to confirm thrombus-device integration and increase the chance of thrombus removal.

## Technical report

In our experience with mechanical thrombectomy, we observed that immediate pre-pull runs showing flow were not returning thrombus and not resulting in recanalization.

Informed patient consent was obtained from all patients described in the four cases below. No identifying patient information is included in this report.

### Case 1

**Figure 1:**
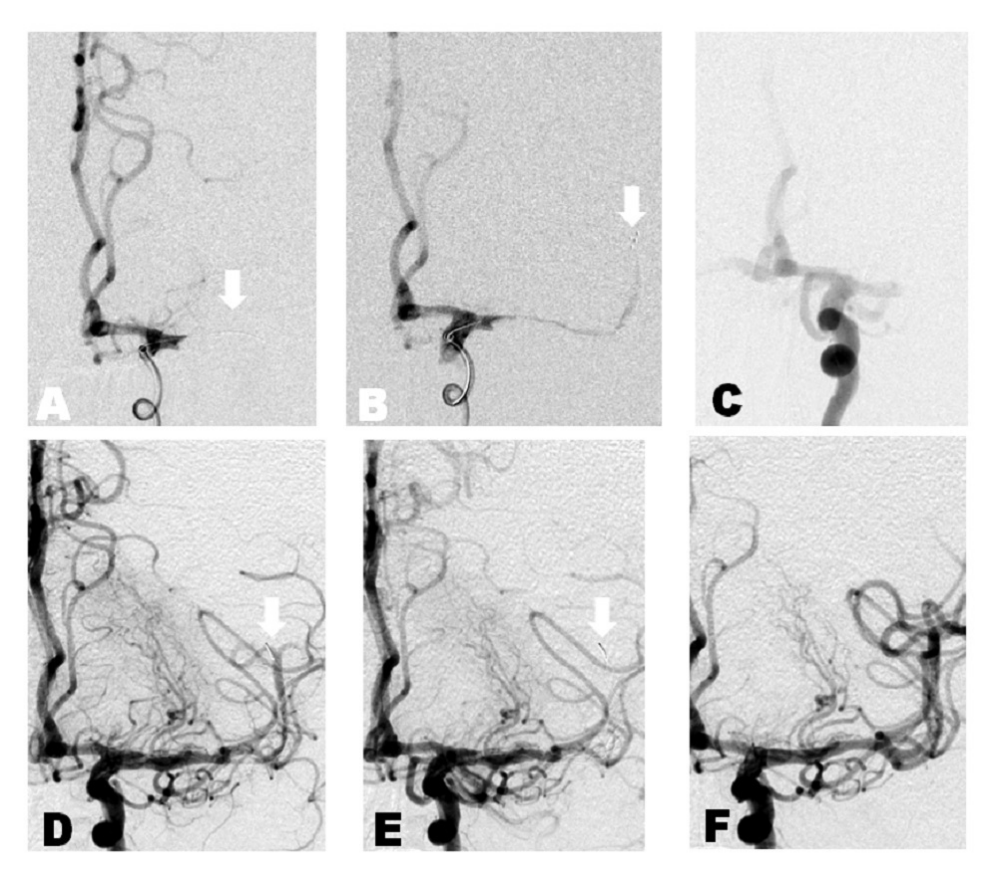
Case 1 A. Anterior-posterior projection digital subtraction angiogram of the left internal carotid artery. White arrow: microcatheter across occluded M1 segment. B. White arrow: flow through stent-retriever immediately before removal of the device. C. Follow-up angiogram reveals persistent occlusion. D. Stent retriever deployed across lesion a second time. White arrow: flow immediately -post deployment. E. White arrow: no flow immediately before removal, possibly suggesting maximized device-thrombus integration. F. TICI Grade 3 recanalization.

A patient in the sixth decade of life was found to have the acute onset of right hemiplegia and global aphasia while on heparin for carotid dissection. Non-invasive imaging showed a complete occlusion of the left M1 segment of the middle cerebral artery and the National Institutes of Health Stroke Scale (NIHSS) was 20 (Figure 1). A Solitaire™ stent retriever was placed across the clot two hours after symptom onset. Flow was noticed through the stent. The device was pulled after a five minute seating time with persistent occlusion seen on follow-up angiogram. Next, a Trevo® was placed due to damage to the Solitaire™ and trickle flow was noticed. A digital subtraction run after five minutes seating time revealed occlusion of the stent. A thrombectomy was performed and the subsequent angiogram showed a thrombolysis in cerebral infarction (TICI) 3 result. At three months post-procedure follow-up, the patient's Modified Rankin Score (mRS) was 3.

### Case 2

**Figure 2:**
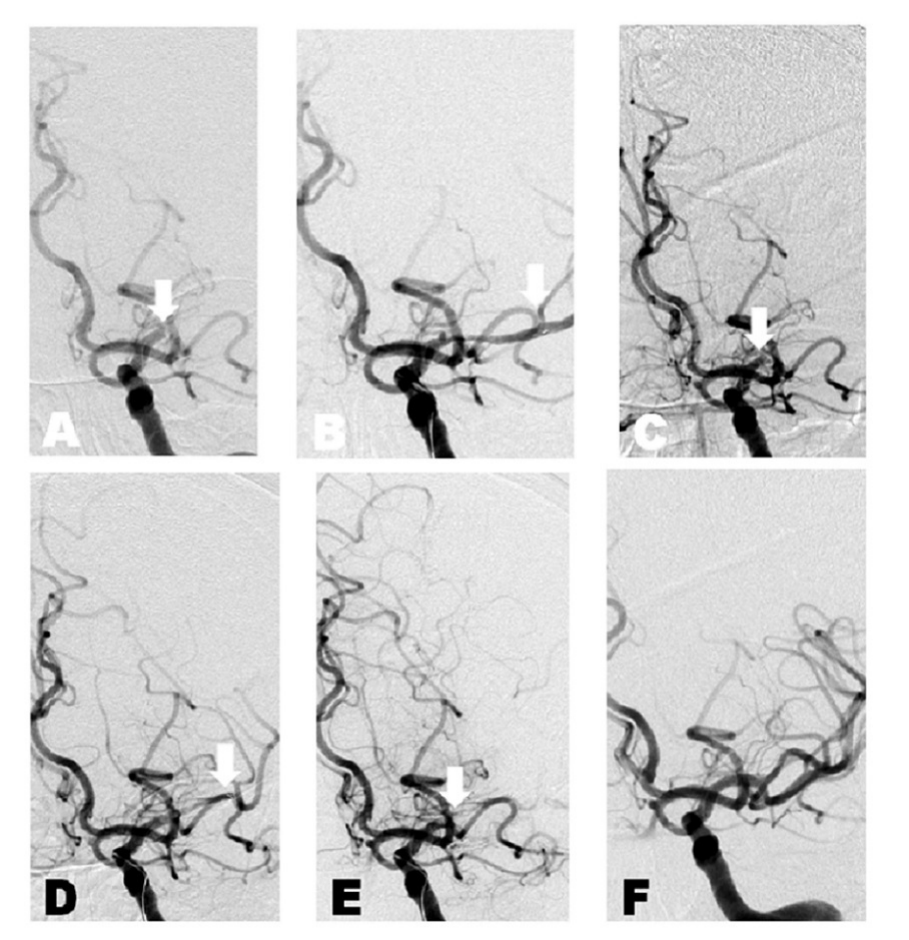
Case 2 A. Anterior-posterior projection digital subtraction angiogram of the left internal carotid artery. White arrow: occlusion of the M1 segment. B. White arrow: flow through stent retriever immediately before removal of the device. C. Follow-up angiogram reveals persistent occlusion. D. Stent retriever deployed across lesion a second time. White arrow: flow immediately post-deployment. E. White arrow: no flow immediately before removal, possibly suggesting maximized device-thrombus integration. F. TICI Grade 3 recanalization.

A patient in the eighth decade of life on Coumadin for a mechanical heart valve presented to the emergency department with the acute onset of aphasia and right hemiplegia. NIHSS was 18. CT angiogram showed clot involving the left supraclinoid internal carotid artery (ICA) and M1 segments (Figure 2). A Solitaire™ device was placed across the clot two hours after symptom onset. After five minutes seating time, a digital subtraction run showed moderate flow and the device was removed. Follow-up angiogram showed TICI 0 result. The Solitaire™ was replaced across the clot 10 minutes later with moderate flow noted again. Immediate pre-removal angiogram at three minutes showed no flow and thrombectomy was performed. Follow-up angiogram showed a TICI 3 result. At three months postop, the patient has achieved mRS 2.

### Case 3

**Figure 3:**
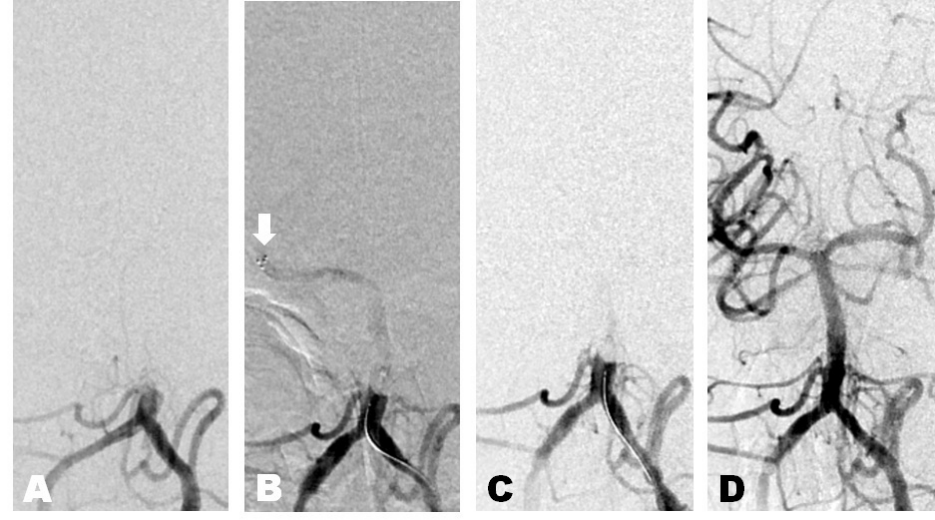
Case 3 A. Anterior-posterior projection digital subtraction angiogram of the left vertebral artery demonstrating an occluded basilar artery. B. White arrow: flow through stent retriever immediately after the device deployment. C. No flow immediately before removal, possibly suggesting maximized device-thrombus integration. F. TICI Grade 3 recanalization.

A patient in the third decade of life presented with the acute onset of left hemiparesis and blurry vision. The NIHSS was 12. Non-invasive imaging showed basilar occlusion (Figure 3). A Solitaire™ device was placed across the clot 10 hours after symptoms onset. A digital subtraction run showed trickle flow. Three minutes after placement, a follow-up run showed no distal flow. A thrombectomy was performed and a subsequent angiogram showed a TICI 3 result. The patient's mRS was 0 at three months.

### Case 4

**Figure 4:**
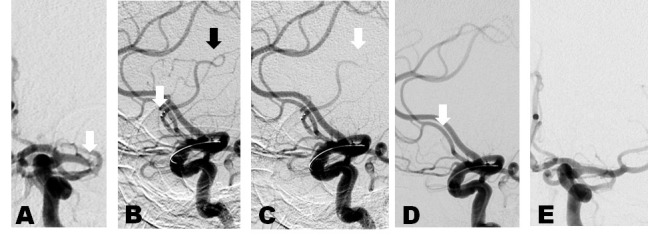
Case 4 A. Anterior-posterior projection digital subtraction angiogram of the left internal carotid artery. White arrow: occlusion of the M1 segment. B. Lateral projection digital subtraction angiogram of the left internal carotid artery. White arrow: stent tines immediately after deployment. Black arrow: flow through stent immediately after deployment. C. Follow-up angiogram at 5 minutes of dwell time. White arrow: persistent flow, although less than in the previous run. D. Follow-up angiogram at 8 minutes of dwell time. White arrow: no flow through the stent. Stent pulled as a result of this angiogram. E. TICI Grade 3 recanalization.

A patient in the eighth decade of life presented after the acute onset of global aphasia and right hemiplegia. NIHSS was 22, and non-invasive imaging showed a complete left M1 occlusion (Figure 4). TPA was administered without effect. A Solitaire™ device was placed across the clot at 5 hours and 40 minutes after the onset of symptoms. Immediate digital subtraction run showed persistent flow. Another angiogram three minutes later showed some flow, although less than the initial post-deployment angiogram. Therefore, the device was allowed to dwell for three more minutes. The next follow-up angiogram revealed no flow through the stent, and a thrombectomy was performed. The post-thrombectomy angiogram showed a TICI 3 result. Unfortunately, the patient suffered a large basal ganglia hemorrhage the following day. Despite aggressive care, the patient’s clinical picture did not improve, and the family elected to withdraw care.

The illustrative cases above allowed us to formulate the appropriate seating time prior to pulling the device. The stent retriever is deployed into the clot per guidelines. A digital subtraction run is then performed looking for flow through the stent into the previously occluded distal vasculature. Flow is usually seen. We allow the device to seat in place for three minutes. Another digital subtraction run is performed. If a flow is present, we continue to allow the device to seat, performing digital subtraction runs every two to three minutes until the flow has stopped. Once the flow has stopped, we perform the thrombectomy. We believe cessation of flow is due to the maximal integration of the clot into the stent cells, providing higher clot surface area for device attachment and a greater likelihood of successful thrombectomy.

## Discussion

Early recanalization of the occluded vasculature is associated with improved outcomes and is the goal of therapy in ischemic stroke [11]. A renaissance of acute stroke care began in 1999 with the PROACT II trial [12]. Patients with middle cerebral artery (MCA) occlusion within six hours of presentation without hemorrhage or infarct on computed tomography (CT) scan were randomized to receive intra-arterial (IA) thrombolytics versus placebo. More good outcomes (mRS ≤ 2) were seen at 90 days with IA therapy versus placebo. Significantly superior recanalization was seen with IA therapy than with placebo, 66% and 18%, respectively. 

The first generation mechanical thrombectomy devices were introduced in 2004: MERCI® (Concentric Medical, Fremont CA) and Penumbra™ (Penumbra, Alameda CA). MERCI I was a case series safety study utilizing the MERCI® device in patients with IV-tPA contraindication and NIHSS ≥ 10 within eight hours of symptoms. Thirty patients were enrolled [13]. Recanalization was seen with the device only in 43% and the device, plus IA-tPA, in 64%. The prospective, single-arm MULTI MERCI trial comparing MERCI® to PROACT II data followed soon thereafter [14]. Recanalization was seen in 57.3% with MERCI® alone and 69.5% with combination IA-tPA. MRS ≤ 2 was achieved in 36% with a mortality of 34%. No outcome measure was significantly superior, but all trended towards significance encouraging further investigation. The Penumbra Pivotal Stroke Trial was the first large device trial to evaluate efficacy in documented large vessel occlusive disease [3]. This was a prospective, single-arm evaluation of the Penumbra™ device in 125 patients with large vessel occlusion within eight hours of symptom onset and NIHSS ≥ 8. In this study, TIMI 2-3 (thrombolysis in myocardial infarction) recanalization was achieved in 81.6% (54.4% TIMI 2; 27.2% TIMI 3), 90-day mRS ≤ 2 in 25% with a 12.8% complication rate, and an all-cause mortality of 32.8%. The outcome data shows a distinct advantage with better recanalization. Patients with TIMI 2-3 results had significantly better rates of discharge NIHSS or improving from pre-treatment by 10 points and 30-day mRS ≤ 2 compared with those with TIMI 0-1.

The IMS III trial attempted to bring a higher grade of evidence to the question of endovascular therapy for stroke. This randomized, controlled, blinded trial of IV-tPA versus IV-tPA and endovascular therapy enrolled patients, aged 18-82, within three hours of symptom onset with NIHSS > 10, although a later exemption was made for NIHSS 8-9 with documented MCA occlusion [11]. Devices included MERCI®, Penumbra™, EKOS®, and, in a small percentage, Solitaire™. The study was stopped for futility after enrollment of 656 patients. Modified Rankin Score of ≤ 2 at 90 days was achieved in 40.8% and 38.7% of patients in the endovascular versus t-PA monotherapy groups, respectively. The 90-day mortality in the groups was 19.1% versus 21.6%, and symptomatic intracranial hematoma (ICH) was 6.2% and 5.9%. While none of this achieved significance, examination of the recanalization data shows promise. TICI 2 or 3 recanalization rates were achieved in 65% of ICA occlusions, 81% of M1, 70% of M2, and 77% of distal M2 occlusions. Of crucial importance was a correlation between recanalization rates and patients who achieved mRS ≤ 2 at 90 days. Modified Rankin Score of mRS ≤ 2 at 90 days was achieved in 12.7% of patients with TICI 0, 27.6% of TICI 1, 34.3% of TICI 2a, 47.9% of TICI 2b, and 71% of TICI 3.

MR-RESCUE was a randomized controlled trial evaluating whether favorable penumbral patterns could identify patients who would benefit from endovascular therapy [14]. One hundred and eighteen patients with large vessel anterior circulation acute ischemic stroke within eight hours of presentation and NIHSS scores 6-29 underwent imaging to determine the presence or absence of a favorable penumbral pattern as determined by study design. They were then randomized to either interventional management with first generation devices or IV-tPA. No significant difference was seen in the 90-day mean mRS, rates of mRS ≤ 2, or late revascularization in either endovascular or medical management arms in either favorable or non-favorable pattern cohorts. Limitations included enrollment of patients with large infarct cores and late time to endovascular therapy. However, most striking were the recanalization numbers. Early recanalization was only measured in the endovascular group and TICI 2b/3 was only achieved in 27%. This is lower than in first and second generation device studies citing 40-45% and 60-80%, respectively [3-4, 9-10]. Looking at patients in both favorable and non-favorable penumbra groups who achieved revascularization, the mean mRS score in patients who were recanalized was significantly better than those who were not (3.5 [3.1 - 3.9] versus 4.4 [4.0 - 4.8], p = 0.04). This again highlights the value of recanalization despite the overall outcome limitations.

While indications and contraindications for endovascular therapy for ischemic stroke remain to be clearly defined, the benefit of early and prompt recanalization is evident. During many interventions, we noticed that many of our pulls were unsuccessful when flow was present through the Solitaire™ device on the pre-pull angiogram. Unsuccessful pulls required replacement of both microcatheter and the interventional device, both of which added time, especially in patients with complicated vasculature. Cumulative time needed to replace the device could be significant in some patients, negatively affecting their outcome. After our observation that an occluded vessel post-device deployment had a positive prediction towards a successful pull, we noticed that our rate of false pulls had decreased. Waiting until vessel occlusion is achieved may prolong time to pull; however, it takes less time than device replacement. In Case 4 above, the total seating time was eight minutes where replacing the device would be 10 minutes, plus another five minutes for seating. Additionally, if the device is occluded prior to the full five minute seating time, then perhaps the thrombectomy can be done earlier, as in Case 3.

This series does not provide enough data to form the basis for a change in technique for all users. Moreover, if a surgeon or interventional team have radiographic success rates higher than those published in trials, they should certainly continue to utilize the techniques that are working for them. The purpose of this manuscript is to share our observations on what techniques might increase the chance of success, especially in cases in which one or perhaps two deployment cycles have failed. Furthermore, we hope to encourage thought and debate about how to best use the tools available to us in the treatment of acute stroke. 

## Conclusions

As mechanical thrombectomy technology advances, interventionalists should fine tune skills to optimize outcomes. Our series has shown that waiting until the flow is absent through the seated stent retriever can increase successful thrombectomy and decrease the time to recanalization. We believe this technique will make placement of a stent retriever more efficient and could lead to better outcomes due to decreased time to recanalization. We hope that the sharing of observations such as this will advance our ability to deliver the best care to stroke patients.
